# Personals

**Published:** 1912-09

**Authors:** 


### PERSONALS.

---

Dr. A. L. Benedict announces the removal of his professional office as well as the headquarters of the BUFFALO MEDICAL JOURNAL, from 354 Franklin St. to the Frontenac, corner of Elmwood Ave. and Summer St., Buffalo.

---

Major John D. Howland, M. D., Captain E. L. Beebe, M. D., First Lt. Arthur C. Schaefer, M. D., and Capt. J. M. O'Gorman (the last named being one of our advertisers) were detailed from Buffalo National Guardsmen for service at the Connecticut manœuvres in August.

---

Dr. J. J. Finerty of Buffalo, was in Detroit early in August.

---

Dr. E. P. Reimann of Buffalo spent a couple of weeks at Honey Harbor in August.

---

Dr. George F. Cott of Buffalo, attended the International Otological Congress in Boston in August.

---

Dr. Eli H. Long of Buffalo, was at Chautauqua about the first of August.

---

Dr. C. S. Lattin of Buffalo, was visited by burglars early in August. The stolen property was recovered from pawn shops.

---

Dr. H. D. Walker of Buffalo, has moved from 915 Main St. to 141 Elmwood Ave.

---

Dr. Benjamin Jacobson of Buffalo, has located at 1270 E. Ferry St.

Dr. James P. Barr of 1366 Broadway, Buffalo, has had a second misfortune. While convalescent from the fracture of several ribs in an automobile accident, his house was damaged by fire but without serious loss.

---

Dr. U. B. Stein of Buffalo, spent some time at Fall River, Mass., about the first of August.

---

Dr. Wm. M. Mehl and Dr. Edward H. Mehl of Buffalo, sailed for Vienna, Aug. 2, on the Kaiser Wilhelm der Grosse.

---

Dr. Marie Ross Wolcott spent the summer traveling in Canada.

---

Dr. William M. Decker of Buffalo, spent August at E. Aurora.

---

Dr. J. Hudson Grant of Albany, formerly of Buffalo, spent August at Revere Beach near Boston.

---

Dr. George A. Himmelsbach of Buffalo, left about the middle of August for a short European trip.

---

Dr. F. A. Kittinger of Lockport, returned in August from Nova Scotia.

---

Dr. Regina Flood Keys of Buffalo, expects to leave home next month for a tour around the world.

---

Dr. Mabel Flood who graduated at the University of Buffalo in 1911, has begun practice with her uncle, Dr. Henry Flood, at 401 Lake St., Elmira.

---

Dr. William Brady of Elmira, visited his parents in Canandaigua, about Aug. 1.

---

Dr. Matthew D. Mann of Buffalo, spent August in the Adirondacks.

---

Dr. Joseph Brennan and Dr. Louis Hengerer, former Buffalonians, now of New York City, visited in Buffalo during August.

---

The house of Dr. J. F. MacPherson of N. Tonawanda, was entered by burglars in the night of Aug. 6-7, while the family were absent from home, Dr. MacPherson having been called to Grimsby, Ont., on account of the serious sickness of his brother. Grimsby is having a typhoid epidemic.

Dr. Myrtle A. Hoag announces the removal of her office from 353 Dearborn St. to 892 West Ave., near Ferry St., Buffalo.

---

Dr. L. G. Hanley of Buffalo, has returned from a month's vacation at his summer home, Woodmont, Ct., and Norfolk, Va.

---

Dr. Alfred Hull Clarke of Buffalo, returned Aug. 1 from a vacation of several weeks at Lake View.

---

Dr. C. T. Crance and Dr. C. W. Clendenan of N. Tonawanda, had a ten-day automobile trip to Crooked Lake, late in July

---

Dr. Jacob Goldberg of Buffalo moved early this summer to 486 Elmwood Ave.

---

Dr. F. C. Busch of Buffalo, sails for Europe Sept. 7, for six months study. Dr. F. C. Pratt will occupy Dr. Busch's residence, on Irving Place.

---

Dr. James W. Charters of Buffalo, made an extended automobile tour through the Thousand Island country and to Ottawa, late in July.

---

Dr. L. H. Staples of Buffalo, spent two weeks on Georgian Bay in July.

---

Dr. William F. Jacobs of Buffalo, was at Lake of Bays for a fortnight in July.

---

Dr. William E. Fogle of Corning was thrown off a street car on July 25, receiving a concussion of the brain. He has been reported as recovering.

---

Dr. Harrison Betts of New York City, visited his old home in Buffalo this summer.

---

Dr. Chauncey P. Smith of Buffalo, traveled in northern Canada during August.

---

Dr. John F. Fairbairn of Buffalo, arrived at Montreal from England, Aug. 11, and proceeded to Boston to attend the meeting of the International Otological Congress, before returning to Buffalo.

---

Dr. and Mrs. Montgomery E. Leary of Rochester, have just returned from the Bermudas.

Dr. and Mrs. F. B. Maynard of Rochester, have returned from a trip to the Massachusetts coast.

---

Dr. W. M. Brown of Rochester, is on a trip camping in Northern Canada.

---

Dr. L. R. Corman has removed his residence to 1140 Park Ave., Rochester. His office is still at 209 Alexander St.

---

Dr. W. A. Keegan of Rochester, has removed his office from Clinton Ave., South, to Alexander St., corner of Park Ave.

---

Dr. John A. Stapleton has just purchased a house in Alexander St., Rochester.

---

Dr. S. W. Bradstreet has also purchased a house in Alexander St., Rochester.

---

Dr. M. B. Palmer of Rochester, is back from a short vacation.

---

Dr. William F. French, Rochester, has returned from an automobile trip to Chicago and return.

---

Dr. W. G. Orchard, Rochester is now occupying his new house in Linden Street.

---

Dr. A. S. Knight, Rochester, has returned from a fishing trip in Canada.

---

Dr. E. B. Angell of Rochester, is away on a vacation in Northern Canada.

---

Dr. C. R. Barber, Rochester, has returned from an automobile tour.

---

Dr. H. M. Imboden, formerly of Clifton Springs Sanitarium and recently of Rochester, has removed to 480 Park Ave., New York.

---

Dr. W. H. Woodbury of Buffalo, is spending the summer in European travel and has mailed to his friends several picture postal cards of views taken by himself.

---

Dr. F. A. Helwig of Akron, has been elected President of the Akron Board of Education.

Dr. Alfred B. Wright of Buffalo, has returned from a summer spent in camp in the mountains and will occupy his new home at 12 Chatham Ave., Nye Park.

Dr. Harry R. Trick of Buffalo, has just returned from a two weeks trip up the lakes and to Rochester, Minn., having acquired information and lost no organs.

Dr. F. A. Wollschlaeger of Buffalo, spent part of July and August in Canada.

Dr. Dewitt H. Sherman of Buffalo, motored along the New England coast and proceeded via Yarmouth to Nova Scotia, in August.

Dr. M. Allen Richter of Buffalo, spent the latter part of August in Maple Leaf, Mich.

Dr. Francis M. O'Gorman of Buffalo has moved to his new home, 92 Blaine Ave.

Dr. George J. Saylin of Buffalo, is completing a new residence and office on Spring St.

Dr. William Ward Plummer of Buffalo, returned Aug 17, from a six weeks' trip through Yellowstone Park and the west.

Dr. C. Spencer Kinney of the Easton (Pa.) Sanitarium, has been appointed Chief of the Consulting Staff on Mental and Nervous Diseases, to the State Homoeopathic Hospital at Middletown, N. Y.

